# Temporal trends in ultrasound utilisation in the radiology department of a tertiary hospital

**DOI:** 10.4102/sajr.v26i1.2449

**Published:** 2022-08-29

**Authors:** Nwabisa Siyeka, Michelle Da Silva, Richard D. Pitcher

**Affiliations:** 1 Department of Medical Imaging and Clinical Oncology, Faculty of Medicine and Health Sciences, Stellenbosch University, Cape Town, South Africa

**Keywords:** trends, ultrasound, utilisation, radiology, tertiary

## Abstract

**Background:**

Little is known about the combined impact of increasing ultrasound usage by clinical disciplines outside radiology and technical advances in other specialised radiological modalities on the role of ultrasound in tertiary-level radiology departments.

**Objectives:**

The aim of this study was to evaluate temporal trends in ultrasound utilisation in a tertiary-level radiology department.

**Method:**

An institutional review board-approved retrospective descriptive study in the radiology department of Tygerberg Hospital (TBH). The nature and number of ultrasound performed in 2013 and 2019 were retrieved from the TBH radiology information system (RIS). These were compared, expressed as a proportion of the overall annual radiology workload and stratified by location (ultrasound suite, interventional suite, mammography suite). Ultrasound suite examinations were analysed by body part and age (0–13 years; > 13 years) and interventional suite workload by procedure.

**Results:**

The overall radiology workload decreased by 8%, reflecting the interplay between decreased plain radiography (–19%) and general fluoroscopy (–0.3%) and increased computed tomography (27%), magnetic resonance (23%) and fluoroscopically guided procedures (22%).

There was a 12% increase in ultrasound utilisation. Ultrasound remained the second most common specialised imaging investigation throughout, after computed tomography. Ultrasound suite services were stable (–1%) representing a balance between decreased abdominal (–22%) and arterial (–16%) scans, and increased musculoskeletal (67%), small part (65%) and neonatal brain scans (41%). There were substantial increases in interventional (90%) and mammography suite (199%) services.

**Conclusion:**

Ultrasound remains a key modality in the tertiary-level radiology department, with an evolving pattern of clinical applications.

## Introduction

Since the first reported clinical use of diagnostic ultrasound more than 60 years ago, the modality has undergone major technical advances, with the development of real-time imaging, improved spatial resolution, Doppler capability, endo-cavity probes and sonographic contrast material. Ultrasound has many characteristics that make it the ideal imaging modality.^1,2^ It is safe, affordable, has no specific infrastructure or radiation protection requirements and can operate on standard electrical power or battery. Machines are robust, mobile, require minimal maintenance and may be portable. The smallest units now fit into the palm of a hand.^3^ The modality is thus very widely available, being used in radiology departments, outpatient clinics, at the bedside, in operating theatres and labour wards of secondary and tertiary-level hospitals. It is also used in rudimentary rural clinics and in roadside emergencies. It can be used repeatedly on the same patient, without radiation risk and results are rapidly available. With appropriate training, ultrasound can be used by a broad spectrum of medical personnel, and competence can be on a sliding scale, from the very basic to highly sophisticated with incremental competence over time.^4^

Ultrasound is not the exclusive domain of the radiologist. It is increasingly being used by other medical disciplines including family physicians, cardiologists, obstetricians, gynaecologists, urologists, nephrologists, neurosurgeons, neonatologists, emergency medicine physicians, vascular surgeons, ophthalmologists, gastroenterologists and critical care physicians, to name a few.^5^

In addition, as healthcare infrastructure and imaging capability are improved at the primary and secondary levels of care, access to ultrasound at such facilities is typically improved, potentially decreasing reliance on ultrasound investigations performed at tertiary hospitals.

In parallel with the technical advances in ultrasound over the past decades, there have been substantial technological developments in other specialised imaging modalities, particularly CT and MR. These advances include faster scan times, decreasing radiation dosages, improved spatial resolution and a dwindling need for sedation. Thus, although the clinical applications of ultrasound are expanding, the same is true for other modalities, making these potentially more attractive diagnostic options than ultrasound in certain clinical settings.^6,7^

Of note, a recently published study of changes over a decade in public sector diagnostic imaging utilisation in the Western Cape Province (WCP) of South Africa (SA) showed that ultrasound had the highest numerical and percentage growth in usage of all imaging modalities. The increased ultrasound usage was largely attributable to enhanced access at the lower levels of healthcare.^8^

This provides the context for this study. To the best of our knowledge, the cumulative impact of the increasing performance of ultrasound by disciplines outside of radiology, the enhanced availability of ultrasound at primary and secondary levels of care and the technological advances together with the burgeoning usage of other specialised imaging modalities such as CT and MR, on ultrasound utilisation in a tertiary-level radiology department has not been evaluated. Such a study is important to ensure appropriate strategic planning, service delivery and teaching in the tertiary environment, where clinicians typically have recourse to the full spectrum of modern imaging modalities. This study aimed to evaluate temporal trends in ultrasound utilisation in a tertiary-level radiology department.

## Methods

This retrospective study was conducted in the radiology department of Tygerberg Hospital (TBH), a 1386-bed main teaching facility of the Faculty of Medicine and Health Sciences of Stellenbosch University in Cape Town, WCP, SA.

Customised searches of the TBH radiology information system (RIS) were conducted to retrieve relevant details of all ultrasound examinations performed by the radiology department during 1 January 2013 – 31 December 2013 and 1 January 2019 – 31 December 2019. Radiology information system data were cross-checked against the department’s manually collated examination statistics and compared for the two periods. Any study other than plain film radiography was considered a specialised investigation. Ultrasound studies were expressed as a proportion of the total annual radiology workload, as recorded in the annual departmental reports for the respective years. All ultrasound examinations performed by clinical departments outside the radiology department were excluded from analysis.

Further analyses stratified ultrasound studies by location: *general* examinations were those performed either in the radiology department’s ultrasound suite or as mobile studies in hospital wards/intensive care units; *interventional* procedures were executed in the radiology department’s angiography/interventional theatre; *breast* studies were completed in the departmental mammography suite. *General* examinations were further analysed by body part and patient age (0–13 years; > 13 years). Interventional workload was analysed by procedure.

### Ethical considerations

This was a retrospective study and therefore had no impact on current patient management. As only the database of the ultrasound records was accessed, no patient details were compromised.

Complete anonymity was maintained by the use of unique study numbers in place of patients’ names or folder numbers. Ethical approval was provided by the Stellenbosch University, National Health Research Ethics Council (NHREC) registration number: REC-130408-012 (HREC1).

## Results

### Tygerberg Hospital diagnostic imaging utilisation

There was an overall 8% decrease in diagnostic imaging workload (191 163 vs 176 812) in the review period. This reflected the interplay between a striking decrease in plain radiography (132 901 vs 107 722; –19%), a modest decline in general fluoroscopy (7903 vs 7725; –0.3%) and substantial increases in CT (22 956 vs 29 248; 27%), MR (4005 vs 4935; 23%) and fluoroscopically guided procedures (5781 vs 7714; 22%). Plain radiography declined from 70% to 61%, whilst the specialised imaging studies increased from 30% to 39% of the departmental workload (Table 1).

**TABLE 1:** Ultrasound utilisation at Tygerberg hospital.

Ultrasound utilisation at TBH	2013		2019		Total increase	% increase
	*n*	%	*n*	%		
**TBH radiology services**						
**Modality**						
Plain radiography	132 901	70	107 722	61	−25 179	−19
CT	22 956	12	29 248	17	6292	27
Ultrasound	13 594	7	15 282	8	1688	12
General fluoroscopy	7903	4	7725	4	−178	−2
Mammography	4023	2	4186	2	163	4
MR	4005	2	4935	3	930	23
Flouroscopically guided interventional procedures	5781	3	7714	4	421	22
Total	191 163	-	176 812	-	−14 351	−8
**TBH ultrasound services**						
**Investigation**						
General ultrasound	12 256	90	12 155	80	−101	−1
Ultrasound-guided interventions	797	6	1511	10	714	90
Breast ultrasound	541	-	1616	-	1075	199
Total	13 594	-	15 282	-	1687	13
**General ultrasound**						
**Investigation**						
Abdomen	5923	48	4601	38	−1322	−22
KUB	2605	21	3154	26	443	16
Venous Doppler	920	-	983	-	66	7
Arterial Doppler	652	-	550	-	−102	−16
Neonatal brain	573	-	809	-	236	41
Small parts (thyroid, testis, parotid)	463	-	767	-	303	65
MSK	438	-	733	-	294	67
Pericardium	428	-	355	-	−73	−17
Chest and pleura	219	-	200	-	−19	−9
FAST	35	-	3	-	−32	−91
Total	12 256	-	12 155	-	−101	−1
**Age ≤ 13 years**						
**Investigation**						
Abdomen	743	33	690	25	−53	−8
Chest and pleura	62	3	97	3	35	36
FAST	0	0	2	0	2	-
Pericardium	3	0	2	0	−1	−50
KUB	626	28	832	30	206	25
MSK	122	5	206	7	84	41
Neonatal brain	573	26	809	29	236	29
Small parts (thyroid, testis, parotid)	71	3	86	3	15	21
Arterial doppler	3	0	25	1	22	88
Venous doppler	38	2	67	2	32	46
Total	2241	-	2816	-	575	26
**Age > 13 years**						
**Investigation**						
Abdomen	5180	-	3911	-	−1269	−32
Chest and pleura	157	-	103	-	−54	−52
FAST	35	-	1	-	−34	−3400
Pericardium	425	-	353	-	−72	−20
KUB	1979	-	2322	-	237	10
MSK	316	-	527	-	211	40
Parotid	18	-	31	-	13	42
Testes	193	-	280	-	87	31
Thyroid	181	-	370	-	189	51
Arterial doppler	882	-	525	-	−357	−68
Venous doppler	649	-	916	-	267	29
Total	10 015	-	9339	-	−676	−7
**Mobile studies**						
**Investigation**						
Abdomen	340	37	271	22	−69	−20
Neonatal brain	245	26	524	42	279	114
KUB	122	13	205	17	83	68
Chest and pleura	75	8	71	6	−4	−5
Pericardium	72	8	77	6	5	7
Venous doppler	68	7	84	7	16	10
FAST	11	1	1	0	−10	−90
Total	932	-	1233	-	301	32
**Ultrasound-guided interventions**						
**Investigation**						
Aspiration	41	-	214	-	173	422
Arteriography	0	-	124	-	124	-
Liver biopsy	40	-	65	-	25	63
Kidney biopsy	159	-	156	-	−3	−2
Cholangiogram - PTC	61	-	93	-	32	52
Cholangiogram - Stent	24	-	28	-	4	16
Hydatid saline	-	-	4	-	-	-
JJ removal	4	-	-	-	-	-
Nephrostogram	106	-	201	-	95	90
Nephrostomy	156	-	258	-	102	65
Pigtail drainage	206	-	368	-	162	79
Total	797	-	1511	-	714	90
**Breast ultrasound**						
**Investigation**						
Diagnostic studies	525	-	1523	-	998	190
Ultrasound guided breast interventions	16	-	94	-	78	487
Total	541	-	1616	-	1075	199

TBH, Tygerberg Hospital; KUB, kidney, ureter, and bladder; MSK, musculoskeletal; FAST, focused assessment with sonography in trauma; PTC, percutaneous transhepatic cholangiogram; JJ, double J stent.

### Tygerberg Hospital ultrasound utilisation

During the review period the ultrasound personnel and equipment resources remained stable. Ultrasound remained the second most common specialised imaging investigation throughout the review period, after CT. There was a 12% overall expansion of ultrasound utilisation (13 594 vs 15 282), with ultrasound increasing from 7% to 8% of the overall radiological workload. However, there were distinct trends in the various components of the sonographic service. Utilisation of *general ultrasound* declined slightly (12 256 vs 12 155; –1%), whilst there were striking increases in ultrasound*-guided interventional procedures* (797 vs 1511; 90%), *diagnostic breast sonography* (525 vs 1523; 190%) and ultrasound*-guided breast interventions* (16 vs 93; 490%) (Table 1).

### General ultrasound

#### Overview

There was declining use of abdominal (5923 vs 4601; –22%), pericardial (428 vs 355; –17%) and arterial (652 vs 550; –16%) studies, whilst musculoskeletal (438 vs 733; 67%), small parts (463 vs 767; 65%) and neonatal brain (573 vs 809; 41%) scans increased substantially. At the start of the review period, abdominal studies constituted almost half of all general ultrasound examinations (*n* = 5923, 48%), decreasing to just over one-third (*n* = 4601, 38%) (Table 1).

#### Utilisation by age

An evolving emphasis on paediatric services was reflected by a 26% increase (2241 vs 2816) in scans on patients aged 0–13 years, compared with a 7% decrease (10 015 vs 9339) in studies on older patients. At the start of the review period, approximately one-fifth (*n* = 2241; 18%) of general ultrasound was performed on patients of 0–13 years, compared with almost a quarter (*n* = 2816; 23%) at the end.

There were differing patterns of service utilisation between the younger and older groups. Patients aged 0–13 years recorded a slight decrease in abdominal scans (–8%; 743 vs 690) but increases in all other investigations, particularly chest, renal, neonatal brain, musculoskeletal small parts and vascular studies. Amongst older patients, abdominal, chest and arterial studies decreased substantially, whilst small parts and musculoskeletal investigations increased significantly. It is noteworthy that renal, small parts and musculoskeletal scans increased in both age groups (Table 1).

#### Mobile studies

Demand for mobile studies increased substantially (932 vs 1233; 32%), with a corresponding increase in the proportion of examinations (8% vs 10%) performed as mobile investigations. In addition, the mobile service evolved, with neonatal brain scans surpassing abdominal scans as the most common investigation. In 2013, neonatal brain scans represented just over one-quarter (245/932; 26%) of all mobile investigations, and in 2019, just less than half (524/1233; 42%). At the start of the review period, 43% (245/573) of neonatal brain scans were performed as mobile investigations, compared with 65% (534/809) at the end (Table 1).

### Ultrasound-guided interventions

Ultrasound-guided procedures almost doubled in the review period (797 vs 1511; 90%). Although the use of ultrasound guidance for renal biopsies was stable, there was increased utilisation of a broad range of other interventional procedures, most notably needle aspiration of collections (41 vs 214, 422%), nephrostograms (106 vs 201; 90%) and pigtail drainages (206 vs 368; 79%). Furthermore, the use of ultrasound guidance for both arterial and venous access angiography was introduced in the review period (Table 1).

### Breast ultrasound

Ultrasound applications in the mammography suite tripled (541 vs 1616; 199%), with substantial increases in both diagnostic studies and sonar guided procedures (Table 1).

## Discussion

To the best of our knowledge, this represents the first analysis, in any healthcare setting, of the evolving pattern of ultrasound services in the radiology department of a large tertiary hospital. As such, it provides insights into the current tertiary-level imaging milieu, affording appreciation of the nuanced interplay between the modern imaging modalities and the current place of ultrasound in the provision of tertiary radiological services. It is thus of potential interest and relevance to imaging personnel, healthcare managers, medical educators, health systems analysts and health economists.

The importance of assessing temporal trends in the utilisation of diagnostic imaging is underscored by a Medline search of the subject yielding more than 2300 manuscripts. Studies to date have either analysed trends in the use of a specific imaging modality in the investigation of a single clinical symptom, assessed trends in the general use of imaging for evaluation of a single clinical symptom or organ system or documented changes in the role of imaging in a clinical environment.^7,9,10,11,12,13,14^ Furthermore, although a Medline search of the role of ultrasound in clinical practice revealed more than 10 000 articles, these focused on the impact of sonography in a specific clinical setting. There has been no analysis of the evolving pattern of ultrasound in service delivery, given the technological advances and changing roles of other imaging modalities. Such an analysis is important because it informs strategic planning and appropriate resource allocation and thus ultimately enhances clinical service delivery. It is also important towards maintaining a relevant and modern teaching platform. The dearth of documentation on the evolving pattern of ultrasound in the modern imaging environment is surprising because it is now more than 60 years since the seminal manuscripts of John Wild and Ian Donald heralded the diagnostic ultrasound era.^1,2^

The importance of analysing temporal imaging trends is further highlighted if one considers that in 1970, 42% of antenatal patients had an X-ray examination, with almost 90% involving abdominal exposure. However, with the increasing availability of obstetric ultrasound services during the 1970s, this figure dropped to 3% by 1980.^15^

The current study has several key findings. Firstly, there were significant changes in tertiary imaging in the relatively short review period, as demonstrated by an almost 20% decrease in the use of plain radiography and substantial increases in the utilisation of the more capital-intensive modalities such as CT (27%), MR (23%) and fluoroscopically guided procedures (22%). This underscores the flux in tertiary imaging and the need for ongoing monitoring of this environment. Secondly, despite the relatively rapid changes in this setting, ultrasound remained the second most common specialised investigation, recording a 12% expansion of services and increasing from 7% to 8% of all departmental investigations. Thirdly, there were striking changes in the ultrasound services provided, with a decline in the demand for general abdominal studies (–22%) but marked increases in ultrasound-guided procedures (90%), breast (199%), musculoskeletal (67%), small part (65%) and neonatal brain scans (41%).

The decreased use of plain film radiography at the tertiary level can be attributed to the improved imaging infrastructure within the WCP of SA. Between 2013 and 2019, there was progressive expansion of plain radiographic services in peripheral facilities, together with the commissioning of a provincial-wide picture archiving and communication system (PACS). The latter allowed any imaging performed at peripheral facilities to be viewed at tertiary hospitals, eliminating the need for repeat investigations.^8^

The 32% reduction in abdominal ultrasound in patients older than 13 years likely represents preferential use of abdominal CT in specific clinical settings. Incremental technological advances in CT over the past decades have resulted in progressive decreases in radiation dose and scan times, in conjunction with increased spatial resolution and the capacity for multiplanar image reconstruction. These advances have entrenched CT as the modality of choice for definitive assessment of abdominal oncology staging, acute abdominal trauma, bowel obstruction, non-specific acute abdominal pain and post-operative complications.^6,7,16,17^ Similarly, technological CT enhancements could have contributed to preferential use of CT angiography over arterial sonography.

The growth in ultrasound-guided procedures reflects increasing clinical traction of so-called ‘interventional ultrasound’, defined as any diagnostic and therapeutic procedure performed under ultrasound guidance. Advances in this field in turn reflect the broader, sequential technological developments in ultrasound. The most significant advantages of sonographic guidance are real-time imaging with excellent anatomic detail including exquisite vessel visualisation, lack of ionising radiation, decreased procedure time, portability and the cost savings inherent in freeing up the CT scanner for diagnostic examinations.^18^ Berlyne reported the first sonographically guided intervention as early as 1961, utilising a single element transducer to facilitate renal biopsy.^19^ By the early 1970s percutaneous ultrasound-guided biopsies utilising static images were increasingly reported.^18^ In the 1970s the advent of real-time B-mode machines enabled continuous monitoring of needle tip advancement into a predetermined target.^18^ In the 1980s multiple ultrasound-guided drainages and catheterisation procedures such as nephrostomy, paracentesis, cholecystostomy, abscess drainage and vascular catheterisation were established as rapid and safe alternatives to conventional drainage methods and open surgery.^18,20,21,22,23^ A further 1980s milestone was the emergence of colour Doppler imaging whereby blood flow was visualised in 2-D within a colour box displaying the axial motion. In the next two decades, the clinical diffusion of high-resolution small-part transducers facilitated ultrasound-guided percutaneous biopsy of the head and neck, parathyroid, thyroid and lymph nodes.^24,25,26,27^ The marked increase in TBH percutaneous needle aspirations and pigtail drainages (247 vs 582; 136%) underscores the growing realisation that these procedures provide a safe and effective alternative to operative treatment of a broad spectrum of abdominal conditions, including post-operative sepsis, diverticular abscess, complicated appendicitis, liver abscess and abdominal wall collections.^28,29^

The increased recourse to musculoskeletal ultrasound (MSUS) reflects the global trend of broadening sonographic applications in orthopaedics and rheumatology. This is shown by the increase in annual MSUS publications from seven in 1991 to 175 in 2011.^30^ The first documented clinical use of MSUS was half a century ago. McDonald and co-workers in San Diego used an early B-mode unit to differentiate a Baker’s cyst from thrombophlebitis.^31^ The enhanced use of MS ultrasound has been facilitated by the evolution of high-resolution linear probes and the emergence of Power Doppler. The latter was first described by Rubin in 1994 and in the same year its potential for the assessment for synovitis was reported by Newman.^32,33^ Sonar’s capacity for real-time dynamic imaging makes it a powerful tool that is increasingly used in the diagnosis of osteitis, synovitis, synovial hypertrophy, joint effusion, bone erosion, tenosynovitis, enthesitis, other tendon pathology and bursitis. It is also used for interventional guidance, to monitor treatment response and for dynamic evaluation of impingement, subluxation and dislocation.

Breast ultrasound was first used in the 1970s to distinguish solid from cystic breast masses. It is now entrenched as complementary to mammography in the diagnostic setting. The addition of ultrasound to mammography for breast mass evaluation improves specificity and reduces unnecessary biopsies. Ultrasound has proven particularly useful in detecting clinically and mammographically occult cancers in women with dense breasts.^34^ Globally, as reflected in our data, ultrasound-guided breast interventional procedures have increased in volume in recent years and ultrasound guidance is evolving as the primary mode of biopsy.

The main growth in small part scanning is in thyroid sonography, reflecting the recognition that ultrasound is the modality of choice for initial characterisation of thyroid nodules. Since 2012, the American College of Radiologists (ACR) has refined, through several iterations, an ultrasound-based risk stratification system for the identification of thyroid nodules warranting biopsy or sonographic follow-up. The resultant ACR Thyroid Imaging Reporting and Data System (TIRADS) lexicon is now widely accepted and has informed TBH sonographic practice.^35^

A strength of this study was the use of robust RIS data, correlated with manually collated departmental workload statistics, thereby ensuring the integrity of results. An additional strength is that our findings are potentially broadly applicable to tertiary institutions. A limitation was the failure to include clinical data in the analysis. This precluded identification of evolving clinical referral patterns to explain ultrasound utilisation trends, particularly the decreased usage of abdominal and arterial sonography. Future work in this domain will be strengthened by the inclusion of a clinical component to the analysis.

## Conclusion

This study underscores the continued importance of ultrasound services provided by the tertiary-level radiology department and highlights the evolving pattern of such services.

## References

[1] Wild JJ, Reid JM. Diagnostic use of ultrasound. Br J Phys Med. 1956;19(11):248–257;passim. 13404208

[2] Donald I, Macvicar J, Brown T. Investigation of abdominal masses by pulsed ultrasound. Lancet. 1958;271(7032):1188–1195. 10.1016/S0140-6736(58)91905-6 13550965

[3] Kavic MS. The case for ultrasound. J Soc Laparoendosc Surg. 1997;1(2):101–102. PMC3021265 9876655

[4] Pitcher RD. The role of radiology in global health. In: Mollura D, Culp M, Lungren M, editors. Radiology in global health: Strategies, implementation, and applications. Cham: Springer, 2019; p. 157–174. 10.1007/978-3-319-98485-8_14

[5] Brindle HE, Allain TJ, Kampondeni S, et al. Utilization of ultrasound in medical inpatients in Malawi. Trans R Soc Trop Med Hyg. 2013;107(7):405–410. 10.1093/trstmh/trt034 23764740

[6] Smith-Bindman R, Miglioretti DL, Larson EB. Rising use of diagnostic medical imaging in a large integrated health system. Health Aff. 2008;27(6):1491–1502. 10.1377/hlthaff.27.6.1491 PMC2765780 18997204

[7] Moreno CC, Hemingway J, Johnson AC, Hughes DR, Mittal PK, Duszak R. Changing abdominal imaging utilization patterns: Perspectives from medicare beneficiaries over two decades. J Am Coll Radiol. 2016;13(8):894–903. 10.1016/j.jacr.2016.02.031 27084072

[8] Van Wijk M, Barnard MM, Fernandez A, Cloete K, Mukosi M, Pitcher RD. Trends in public sector radiological usage in the Western Cape province, South Africa: 2009–2019. S Afr J Radiol. 2021;25(1):a2251. 10.4102/sajr.v25i1.2251 PMC8661274 34917410

[9] Smith L, Barratt A, Buchbinder R, Harris IA, Doust J, Bell K. Trends in knee magnetic resonance imaging, arthroscopies and joint replacements in older Australians: Still too much low-value care? ANZ J Surg. 2020;90(5):833–839. 10.1111/ans.15712 32062868

[10] Al Badarin FJ, Chan PS, Spertus JA, et al. Temporal trends in test utilization and prevalence of ischaemia with positron emission tomography myocardial perfusion imaging. Eur Heart J Cardiovasc Imaging. 2020;21(3):318–325. 10.1093/ehjci/jez159 31292618 PMC7778342

[11] Gyftopoulos S, Harkey P, Hemingway J, Hughes DR, Rosenkrantz AB, Duszak R. Changing musculoskeletal extremity imaging utilization from 1994 through 2013: A medicare benefciary perspective. Am J Roentgenol. 2017;209(5):1103–1109. 10.2214/AJR.17.18346 28777654

[12] Braga JR, Leong-Poi H, Rac VE, Austin PC, Ross HJ, Lee DS. Trends in the use of cardiac imaging for patients with heart failure in Canada. JAMA Netw Open. 2019;2(8):e198766. 10.1001/jamanetworkopen.2019.8766 31397858 PMC6692835

[13] Pakpoor J, Raad M, Harris A, et al. Diagnostic imaging use for the initial evaluation of low back pain by primary care providers in the United States: 2011–2016. J Am Coll Radiol. 2019;16(11):1522–1527. 10.1016/j.jacr.2019.04.015 31125539

[14] Juliusson G, Thorvaldsdottir B, Kristjansson JM, Hannesson P. Diagnostic imaging trends in the emergency department: An extensive single-center experience. Acta Radiol Open. 2019;8(7):1–6. 10.1177/2058460119860404 PMC6669846 31392034

[15] Gordon A, Pinchen C, Walker E, Tudor J. The changing place of radiology in obstetrics. Br J Radiol. 1984;57(682):891–893. 10.1259/0007-1285-57-682-891 6487959

[16] Nelms DW, Kann BR. Imaging modalities for evaluation of intestinal obstruction. Clin Colon Rectal Surg. 2021;34(4):205–218. 10.1055/s-0041-1729737 34305469 PMC8292005

[17] Pickhardt PJ, Nelson L. Acute non-traumatic abdominal pain by quadrant: Relative yield of CT and clinical evaluation for diagnosis in 1000 patients. Abdom Radiol. 2019;44(9):2963–2970. 10.1007/s00261-019-02064-6 31104074

[18] Nielsen MB, Søgaard SB, Bech Andersen S, et al. Highlights of the development in ultrasound during the last 70 years: A historical review. Acta Radiol. 2021;62(11):1499–1514. 10.1177/02841851211050859 34791887

[19] Berlyne GM. Ultrasonics in renal biopsy an aid to determination of kidney position. Lancet. 1961;278(7205):750–751. 10.1016/S0140-6736(61)90695-X 13867961

[20] Van Sonnenberg E, Ferrucci JT, Mueller PR, Wittenberg J, Simeone JF. Percutaneous drainage of abscesses and fluid collections: Technique, results, and applications. Radiology. 1982;142(1):1–10. 10.1148/radiology.142.1.7053517 7053517

[21] McGahan JP. Aspiration and drainage procedures in the intensive care unit: Percutaneous sonographic guidance. Radiology. 1985;154(2):531–532. 10.1148/radiology.154.2.3966140 3966140

[22] Juul N, Sztuk FJS, Torp-Pedersen S, Burcharth F. Ultrasonically guided percutaneous treatment of liver abscesses. Acta Radiol. 1990;31(3):275–277. 10.1080/02841859009171990 2201328

[23] Meyer-Schwickerath M, Heckemann R, Eickenberg HU, Hartung R. Ultrasound guided percutaneous nephrostomy. Real-time procedure compared to former methods. Acta Urol Belg. 1983;51(4):435–440. 6650291

[24] Elvin A, Sundström C, Larsson SG, Lindgren PG. Ultrasound-guided 1.2-mm cutting-needle biopsies of head and neck tumours. Acta Radiol. 1997;38(3):376–380. 10.1080/02841859709172087 9191427

[25] Karstrup S, Glenthøj A, Torp-Pedersen S, Hegedüs L, Holm HH. Ultrasonically guided fine needle aspiration of suggested enlarged parathyroid glands. Acta Radiol. 1988;29(2):213–216. 10.3109/02841858809174995 2965906

[26] Court-Payen M, Nygaard B, Horn T, et al. US-guided fine-needle aspiration biopsy of thyroid nodules. Acta Radiol. 2002;43(2):131–140. 10.1080/028418502127347880 12010289

[27] Tikkakoski T, Siniluoto T, Ollikainen A, Päivänsalo M, Lohela P, Apaja-Sarkkinen M. Ultrasound-guided aspiration cytology of enlarged lymph nodes. Acta Radiol. 1991;32(1):53–56. 10.1177/028418519103200114 2012731

[28] Renwick I. Postoperative abdominal sepsis: Imaging and percutaneous management. Surgery. 2012;30(12):659–661. 10.1016/j.mpsur.2012.10.010

[29] Harclerode TP, Gnugnoli DM. Percutaneous abscess drainage [homepage on the Internet]. Treasure Island (FL): StatPearls Publishing; 2022 Jan [cited 2022 Mar 3]. Available from: https://pubmed.ncbi.nlm.nih.gov/33232026/. 33232026

[30] Cannella AC, Kissin EY, Torralba KD, Higgs JB, Kaeley GS. Evolution of musculoskeletal ultrasound in the United States: Implementation and practice in rheumatology. Arthritis Care Res. 2014;66(1):7–13. 10.1002/acr.22183 24115730

[31] McDonald DG, Leopold GR. Ultrasound B-scanning in the differentiation of Baker’s cyst and thrombophlebitis. Br J Radiol. 1972;45(538):729–732. 10.1259/0007-1285-45-538-729 5078938

[32] Rubin JM, Bude RO, Carson PL, Bree RL, Adler RS. Power doppler US: A potentially useful alternative to mean frequency-based color Doppler US. Radiology. 1994;190(3):853–856. 10.1148/radiology.190.3.8115639 8115639

[33] Newman JS, Adler RS, Bude RO, Rubin JM. Detection of soft-tissue hyperemia: Value of power Doppler sonography. Am J Roentgenol. 1994;163(2):385–389. 10.2214/ajr.163.2.8037037 8037037

[34] Joe BN, Sickles EA. The evolution of breast imaging: Past to present. Radiology. 2014;273(2S):S23–S44. 10.1148/radiol.14141233 25340437

[35] Tessler FN, Middleton WD, Grant EG, et al. ACR thyroid imaging, reporting and data system (TI-RADS): White paper of the ACR TI-RADS committee. J Am Coll Radiol. 2017;14(5):587–595. 10.1016/j.jacr.2017.01.046 28372962

